# Cadmium-induced ethylene production and responses in *Arabidopsis thaliana* rely on *ACS2* and *ACS6* gene expression

**DOI:** 10.1186/s12870-014-0214-6

**Published:** 2014-08-01

**Authors:** Kerim Schellingen, Dominique Van Der Straeten, Filip Vandenbussche, Els Prinsen, Tony Remans, Jaco Vangronsveld, Ann Cuypers

**Affiliations:** 1 Centre for Environmental Sciences, Hasselt University, Agoralaan Building D, Diepenbeek, 3590, Belgium; 2 Laboratory for Functional Plant Biology, Ghent University, Karel Lodewijk Ledeganckstraat 35, Ghent, 9000, Belgium; 3 Laboratory of Plant Growth and Development, University of Antwerp, Groenenborgerlaan 171, Antwerp, 2020, Belgium

**Keywords:** 1-aminocyclopropane-1-carboxylic acid, acs2-1acs6-1 knockout mutant, Arabidopsis thaliana, Cadmium, Ethylene, Gene expression

## Abstract

**Background:**

Anthropogenic activities cause metal pollution worldwide. Plants can absorb and accumulate these metals through their root system, inducing stress as a result of excess metal concentrations inside the plant. Ethylene is a regulator of multiple plant processes, and is affected by many biotic and abiotic stresses. Increased ethylene levels have been observed after exposure to excess metals but it remains unclear how the increased ethylene levels are achieved at the molecular level. In this study, the effects of cadmium (Cd) exposure on the production of ethylene and its precursor 1-aminocyclopropane-1-carboxylic acid (ACC), and on the expression of the ACC Synthase (*ACS*) and ACC Oxidase (*ACO*) multigene families were investigated in *Arabidopsis thaliana*.

**Results:**

Increased ethylene release after Cd exposure was directly measurable in a system using rockwool-cultivated plants; enhanced levels of the ethylene precursor ACC together with higher mRNA levels of ethylene responsive genes: *ACO2*, *ETR2* and *ERF1* also indicated increased ethylene production in hydroponic culture. Regarding underlying mechanisms, it was found that the transcript levels of *ACO2* and *ACO4*, the most abundantly expressed members of the *ACO* multigene family, were increased upon Cd exposure. ACC synthesis is the rate-limiting step in ethylene biosynthesis, and transcript levels of both *ACS2* and *ACS6* showed the highest increase and became the most abundant isoforms after Cd exposure, suggesting their importance in the Cd-induced increase of ethylene production.

**Conclusions:**

Cadmium induced the biosynthesis of ACC and ethylene in *Arabidopsis thaliana* plants mainly via the increased expression of *ACS2* and *ACS6*. This was confirmed in the *acs2-1acs6-1* double knockout mutants, which showed a decreased ethylene production, positively affecting leaf biomass and resulting in a delayed induction of ethylene responsive gene expressions without significant differences in Cd contents between wild-type and mutant plants.

## Background

Industrial activities and the application of fertilisers, pesticides and sewage sludge in agriculture have contributed to the dispersion of toxic metals, such as cadmium (Cd), in all ecosystem compartments worldwide [1]. Growing on contaminated soils, plants can take up and accumulate Cd through their root system and transport it to the aboveground plant parts [2],[3]. Cadmium bioaccumulation ultimately leads to the introduction of Cd into the food chain, eliciting threats to the public health, even when present at trace concentrations [4]-[6]. Consequently, the reorientation from agricultural to non-food crops is occurring in contaminated areas. These crops are selected for their metal resistance and accumulation capacity, with the final objective to stabilise and clean the soils in a process called phytoremediation [7]-[10].

Cadmium is a highly phytotoxic, non-essential element that reduces plant growth and inhibits photosynthesis. Cadmium-induced phytotoxicity is a result of cellular and molecular interactions such as: (1) inactivating and/or denaturing biomolecules by binding their functional groups, (2) replacing essential elements (co-factors) showing chemical similarities and (3) increasing the production of reactive oxygen species (ROS), hereby affecting the cellular redox state [5],[11]-[14].

Phytohormones are known to be affected by multifarious biotic and abiotic stress conditions (e.g. toxic metals) and play important roles as signal molecules, integrating developmental programs and responses to environmental stimuli [15]-[17]. The gaseous hormone ethylene is involved in multiple molecular, biochemical and physiological processes during the entire life cycle of the plant and has also been related to enhanced ROS accumulation [18],[19]. A relatively simple metabolic pathway controls the biosynthesis of ethylene [20]. Methionine, the biological precursor of ethylene, is converted to S-adenosylmethionine (SAM) by SAM Synthetase. 1-aminocyclopropane-1-carboxylic acid (ACC) Synthase (ACS) uses SAM as a substrate to form ACC. This is mostly the rate-limiting step in the biosynthesis of ethylene. ACC is oxidised to ethylene by ACC Oxidase (ACO), with CO_2_ and cyanide as by-products [20]-[22]. In *Arabidopsis thaliana*, both ACS and ACO are encoded by multigene families, regulated at the transcriptional level by developmental as well as environmental signals [23]-[26]. In addition, ACS proteins can also be post-translationally modified (e.g. phosphorylation), influencing their stability [27].

Ethylene is often considered as the ‘stress hormone’, modulating multiple defense responses to stresses such as wounding, hypoxia, drought and excess ozone or salt but for example also partially controlling mycorrhizal development and colonisation [17],[21],[28]-[31]. It is known that an increasing ethylene production ensued by regular signal transduction can inhibit plant development and accelerate senescence and abscission processes [20],[22],[32]. Hence, a better understanding of the metal-induced effects on the ethylene biosynthesis pathway improves our knowledge on plant metal resistance, which can be implemented in future research concerning the phytoremediation of contaminated soils.

Although the responses of ethylene production of plants to different toxic metals have already been investigated many times, the mechanistic basis remains unclear [15],[17],[33]-[35]. It is indeed well known that the effect of exposure to metals on ethylene production is metal and concentration specific [34]. Mertens et al. [36] observed an increasing ethylene production in 7 days old *Arabidopsis thaliana* plants exposed to 25 – 500 μM copper (Cu) and zinc (Zn) for up to 6 hours. Lequeux et al. [37], on the other hand, did not observe an effect on ethylene production in 9 days old *Arabidopsis thaliana* plants exposed to 50 μM Cu for 24 hours. In addition, Groppa et al. [38] reported that metal-induced effects on ethylene production are also species-specific. A 14 hours exposure to 1 mM of either Cd or Cu increased the ethylene production in 4 weeks old wheat leaves, whereas in sunflower leaves only Cu enhanced the ethylene production. Rodriguez-Serrano et al. [39] detected a higher ethylene production in 14 days old pea plants exposed to 50 μM Cd for 14 days. Exposure to 400 μM Cd or Cu, but not Zn nor nickel (Ni), differently induced ethylene production in various plant parts of *Arabidopsis thaliana*[15]. The effect of these different metals on the ethylene release was also inversely proportional to the age of the plant parts.

Whereas previous studies only investigated the effect of metals on the ethylene production levels, the aim of the present study is to unravel the mechanisms of Cd-enhanced ethylene biosynthesis. Therefore, we characterised the molecular basis of this response in *Arabidopsis thaliana* plants exposed to environmentally realistic Cd concentrations. We hypothesised that Cd induces ethylene biosynthesis through alterations in the expression of genes encoding the ACS enzymes, the rate-limiting step in ethylene biosynthesis, yielding the basis of the Cd-induced ethylene production that may influence acclimation to Cd stress.

## Results

### Biosynthesis of the ethylene precursor ACC in wild-type plants

The immediate precursor of ethylene, ACC, can be reversibly conjugated to malonyl-ACC or γ-L-glutamyl-ACC (MACC, GACC) [40],[41]. In most cases, the presence of ACC reflects the activity of the rate-limiting step in ethylene biosynthesis that eventually determines the hormonal content. In order to evaluate the effect of Cd on ethylene biosynthesis, we first estimated the concentration of free as well as conjugated ACC in wild-type *Arabidopsis thaliana* plants exposed to Cd.

**Figure 1:**
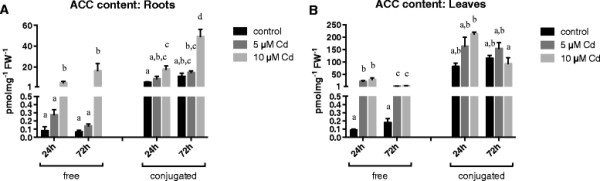
**ACC content.** ACC content (free and conjugated; pmol mg^−1^ FW^−1^) in roots **(A)** and leaves **(B)** of 3 weeks old *Arabidopsis thaliana* plants exposed for 24 or 72 h to either 5 or 10 μM CdSO_4_ or grown under control conditions in a hydroponic culture system. Data are given as mean ± s.e. of at least 5 biological replicates. The letters a-d **(A)** & a-c **(B)** represent groups with significantly different amounts of ACC (Tukey’s test: p < 0.05; except for free ACC content in the roots, Wilcoxon rank sum test: p < 0.05). Statistics was performed separately for free and conjugated ACC.

In roots, exposure to 5 μM Cd had no significant effect on the concentration of either the free or the conjugated ACC (Figure 1A). Exposure to 10 μM Cd on the other hand increased the concentration of both forms of ACC (Figure 1A). While the concentration of free ACC was comparable after 24 h and 72 h of exposure to Cd, the conjugated ACC content continued to increase towards the later time point. The relative impact of Cd on the content of free ACC was higher compared to conjugated ACC (Figure 1A).

In leaves, both concentrations of Cd induced the same significant increase in free ACC, with a maximum content after 24 h of exposure (Figure 1B). Although the abundance of conjugated ACC in general was always higher, the free ACC content was significantly more affected by Cd, whereas conjugated ACC content in the leaves only showed an increasing trend after 24 h of exposure (Figure 1B).

### Gene expression of genes involved in ACC and ethylene biosynthesis

ACC is produced by ACS enzymes, originating from a multigene family. Within this 12-membered family, *ACS3* is a pseudogene and *ACS10* and A*CS12* encode aminotransferases with different functions [24]. This leaves 9 actual *ACS* genes, whose induced expression may contribute to increased ACC synthesis, that were analysed in this study. The expression of *ACS9* was generally below detection limit in our experimental conditions, confirming earlier observations that *ACS9* transcription is nearly absent in vegetative tissues [23]. Transcript levels of *ACS1*, only functional as a heterodimer, were also very low under control conditions. Analysis of gene family expression included quantification of the total transcript abundance of all isoforms together, as well as the relative contribution of each member. Additional file 1A and B shows the relative expression of the individual gene family members to the untreated controls.

**Figure 2:**
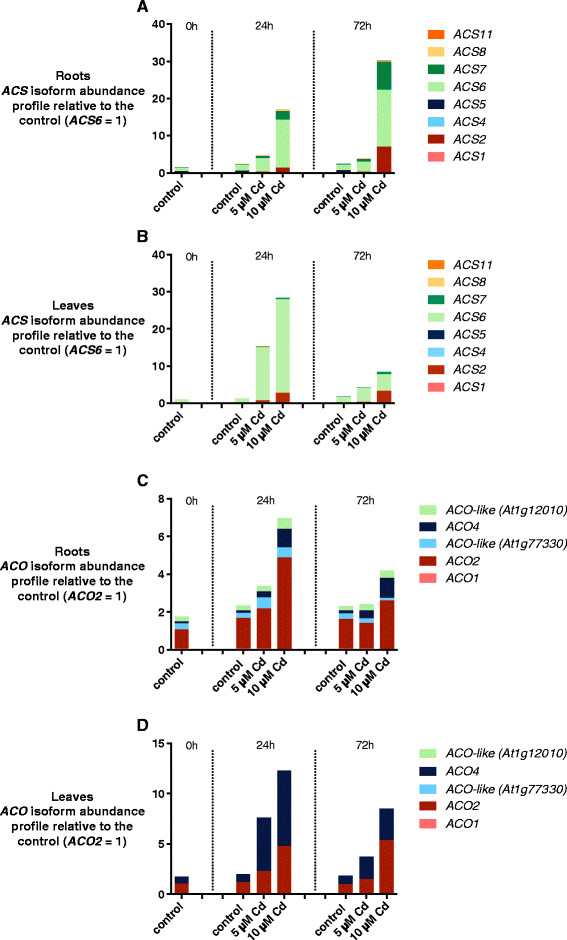
**Relative abundance of*ACS*and*ACO*multigene family.** Relative abundance of *ACS***(A-B)** and *ACO***(C-D)** multigene family members in roots and leaves of 3 weeks old *Arabidopsis thaliana* plants exposed for 0, 24 or 72 h to either 5 or 10 μM CdSO_4_ or grown under control conditions in a hydroponic culture system. Data represent mean abundance of at least 4 biological replicates relative to the control (0 h, 0 μM CdSO_4_) and with the abundance of the most highly expressed family member set at 1 under the control condition. **(A)** Relative abundance of ACS multigene family members in roots. **(B)** Relative abundance of ACS multigene family members in leaves. **(C)** Relative abundance of ACO multigene family members in roots. **(D)** Relative abundance of ACO multigene family members in leaves.

In roots, total *ACS* transcript abundance increased after exposure to Cd in a time- and dose-dependent manner, peaking after 72 h of treatment with 10 μM Cd (Figure 2A). Induction of the transcript levels of *ACS2*, *ACS6* and *ACS7* seemed to particularly contribute to this increased expression level of *ACS* (Figure 2A). Furthermore, the gene expression of *ACS8* also increased significantly after exposure to 10 μM Cd (Additional file 1A), although the relative transcript abundance remained low. In leaves, the highest increase in *ACS* gene expression occurred after 24 h of treatment with Cd (Figure 2B). The transcript abundance of *ACS2* and *ACS6* was mostly affected upon Cd exposure (Figure 2B). Expression of *ACS7* and *ACS8* was also slightly, although significantly upregulated (Figure 2B; Additional file 1B). ACS6 was the isoform with the most abundant transcript levels under control conditions in roots and leaves of *Arabidopsis thaliana*, and was also Cd responsive in both organs (Figure 2B).

In addition, gene expression of the 5-membered *ACO* multigene family, which encodes the proteins catalysing the final step of the ethylene biosynthesis, was also analysed [21],[23],[24].

The rise in total transcript levels of the *ACO* multigene family reached a maximum after 24 h of exposure to Cd. In roots this was mainly due to the Cd-induced *ACO2* expression, however *ACO4* transcript levels also increased after exposure to 10 μM Cd (Figure 2C; Additional file 1A). In leaves, gene expression of both *ACO2* and *ACO4* increased after treatment with 5 or 10 μM Cd (Figure 2D; Additional file 1B). Hence, these were generally the ACO isoforms with the most abundant transcript levels in both organs after Cd exposure.

### Ethylene emission: a comparison between wild-type and *acs2-1acs6-1* mutant plants

The production of ACC by ACS is the rate-limiting step in the ethylene production of *Arabidopsis thaliana*. Our qRT-PCR data suggests that mainly *ACS2* and *ACS6* contributed to the increased expression of *ACS* genes after exposure to Cd. To verify the importance of these genes for Cd-induced ethylene production, wild-type and *acs2-1acs6-1* double knock-out mutant *Arabidopsis thaliana* plants were investigated. First, Cd accumulation was compared between wild-type and mutant *acs2-1acs6-1* plants to assess whether genotypic differences in Cd uptake may be present. In hydroponically cultivated plants, the Cd content in roots and leaves of both genotypes increased in a time- as well as dose-dependent manner (Table 1A). The Cd content in plants treated with 5 μM Cd was similar in roots and leaves. After exposure to 10 μM Cd, roots accumulated twice as much Cd compared to leaves (Table 1A). No significant differences in Cd accumulation were observed between the wild-type and *acs2-1acs6-1* mutant plants.

**Table 1:** **Cadmium content of** ***Arabidopsis thaliana*** **grown in different culture systems**

**A**				
*Cd content*	**Hydroponics**			
	24 h		72 h	
*Roots*	wild-type	*acs2-1acs6-1*	wild-type	*acs2-1acs6-1*
0 μM CdSO_4_	nd	nd	nd	nd
5 μM CdSO_4_	923 ± 16 a	692 ± 54 a	1712 ± 151 a	1327 ± 167 a
10 μM CdSO_4_	3833 ± 449 b	3079 ± 195 b	6465 ± 476 b	5674 ± 633 b
*Leaves*	wild-type	*acs2-1acs6-1*	wild-type	*acs2-1acs6-1*
0 μM CdSO_4_	nd	nd	nd	nd
5 μM CdSO_4_	976 ± 137 a	883 ± 16 a	1527 ± 106 a	1451 ± 32 a
10 μM CdSO_4_	1683 ± 100 b	1829 ± 163 b	2989 ± 335 b	3069 ± 74 b
**B**				
*Cd content*	**Rockwool**			
	24 h		72 h	
*Leaves*	wild-type	*acs2-1acs6-1*	wild-type	*acs2-1acs6-1*
0 μM CdSO_4_	nd	nd	nd	nd
5 μM CdSO_4_	133 ± 7 a	134 ± 8 a	168 ± 18 a	149 ± 41 a
10 μM CdSO_4_	222 ± 16 b	128 ± 37 a	192 ± 30 a	288 ± 48 a

**Figure 3:**
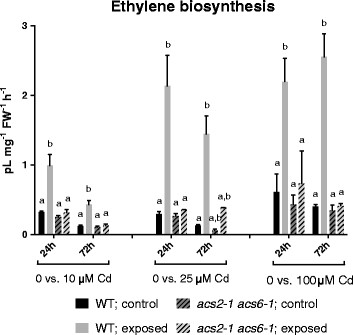
**Ethylene emission.** A comparison of the ethylene emission (pL mg^−1^ FW^−1^ h^−1^) in 3 weeks old wild-type or *acs2-1acs6-1* mutant *Arabidopsis thaliana* plants exposed for 24 or 72 h to 10, 25 or 100 μM CdSO_4_ or grown under control conditions in a rockwool culture system. Data are shown as mean ± s.e. of at least 3 biological replicates. The letters a-b represent groups with a significantly different ethylene production (Tukey’s test: p < 0.05; except for 25 μM CdSO_4_ - 72 h, Wilcoxon rank sum test: p < 0.05). Statistics was performed separately for each Cd concentration and within each exposure time.

The ethylene emission of whole plants was measured as described by Woltering et al. [42], using a rockwool cultivation system. Since Cd uptake in rockwool cultivated plants may differ from that in hydroponics, which in turn may affect ethylene production, both growth systems were also compared for Cd accumulation. Therefore, the internal Cd concentration in the leaves of these plants was compared with the previous results of the hydroponically grown plants (Table 1). Overall, the Cd uptake in rockwool-cultivated plants was six to fifteen times lower compared to hydroponically grown plants. Cadmium accumulation in mutant plants exposed to 5 μM Cd did not significantly differ from the wildtype. On the other hand, 24 h of exposure to 10 μM Cd led to a significantly lower Cd content in the mutant plants, but no significant differences were observed after 72 h of exposure to 10 μM Cd (Table 1B). In order to reach internal Cd concentrations comparable to those attained in hydroponically grown plants, higher external Cd concentrations were applied in the rockwool cultivation system to provoke Cd-induced ethylene production. Consequently, concentrations of 10 μM, 25 μM or 100 μM Cd were applied. Exposure to the various Cd concentrations always significantly increased the ethylene emission in wild-type plants. In the *acs2-1acs6-1* double KO-mutants on the other hand, at none of the applied concentrations a Cd-induced increase in ethylene emission was observed (Figure 3).

As already mentioned, ethylene is a modulator of growth and developmental stages during the entire life cycle of the plant and it is responsible for the induction of cell senescence. Because of the difference in ethylene production between both genotypes, the biomass of roots and leaves was compared after Cd exposure. Furthermore, the growth inhibition caused by exposure to Cd was determined in both organs relative to the controls within each genotype.

**Figure 4:**
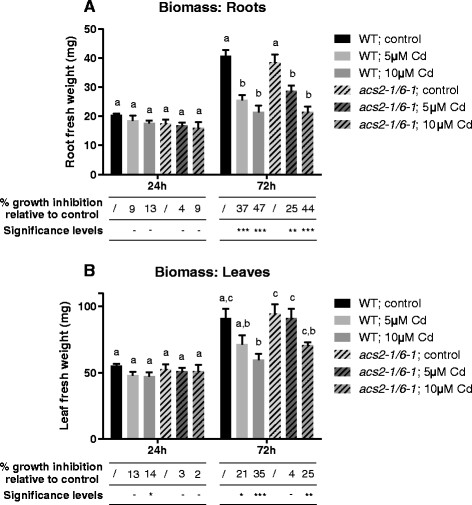
**Biomass & growth inhibition.** A comparison of the fresh weight biomass and growth inhibition (mg) of roots **(A)** and leaves **(B)** of 3 weeks old wild-type or *acs2-1acs6-1* mutant *Arabidopsis thaliana* plants exposed for 24 or 72 h to either 5 or 10 μM CdSO_4_ or grown under control conditions in a hydroponic culture system. Biomass: Data shows mean ± s.e. of at least 4 biological replicates. The letters a-c represent groups with a significantly different biomass (Tukey’s test: p < 0.05). Statistics was performed separately within each exposure time. Growth inhibition: Data shows mean ± s.e. of at least 4 biological replicates relative to the control within each exposure time and genotype. Significance levels: − = no significant difference; * = p < 0.1; ** = p < 0.05; *** = p < 0.01 (Tukey’s test). Statistics was performed separately within each exposure time and genotype.

Neither the wild-type nor the mutant plants showed a significant decrease in root biomass after 24 h of exposure to 5 or 10 μM Cd (Figure 4A). Exposure to either of both Cd concentrations during 72 h did induce a significant reduction in root biomass in both genotypes. The growth inhibition of the Cd-exposed roots relative to the control roots was always higher in the wildtype compared to the *acs2-1acs6-1* mutants (Figure 4A).

In leaves, no significant differences in biomass were observed after 24 h of exposure to 5 or 10 μM Cd between both genotypes. Nevertheless, the growth was significantly inhibited in wild-type plants after 24 h of exposure to 10 μM Cd, which could not be observed in the mutant plants (Figure 4B). Exposure during 72 h to either of both concentrations of Cd did not induce a significant leaf biomass reduction in the *acs2-1acs6-1* mutant plants. On the contrary, there was a significant decrease in leaf biomass of wild-type plants exposed to 10 μM Cd (Figure 4B). Moreover, a significant difference in biomass between the wild-type and mutant plants exposed to 5 μM Cd was observed, which was confirmed by the growth inhibition data. Similar to the roots, the growth inhibition of the leaves in Cd-exposed plants was always higher in the wildtype compared to the *acs2-1acs6-1* mutants (Figure 4B).

### Ethylene responsive genes: a comparison between wild-type and *acs2-1acs6-1* mutant plants

To investigate whether the differences in ethylene production between *acs2-1acs6-1* mutants and wild-type plants were sufficient to provoke a differential ethylene response, expression of primary ethylene responsive genes was measured in both genotypes. The genes encoding for the ethylene receptor *ETR2*, the biosynthesis enzyme *ACO2* and the ethylene response factor *ERF1* are known to be ethylene responsive [22].

**Figure 5:**
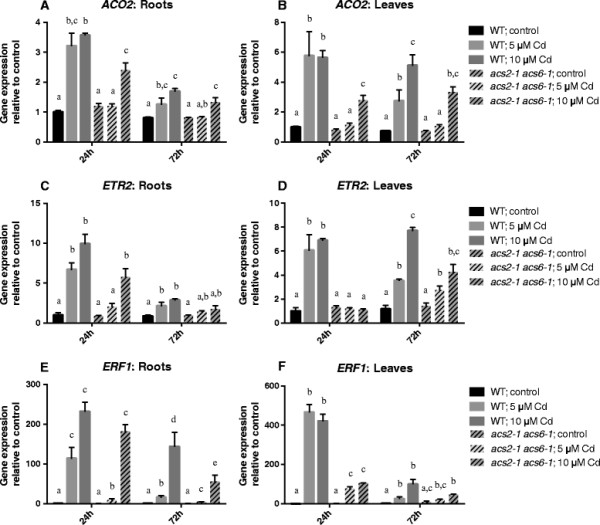
**Relative expression of ethylene responsive genes.** A comparison of the relative expression of *ACO2***(A-B)**, *ETR2***(C-D)** and *ERF1***(E-F)** in roots and leaves of 3 weeks old wild-type or *acs2-1acs6-1* mutant *Arabidopsis thaliana* plants exposed for 24 or 72 h to either 5 or 10 μM CdSO_4_ or grown under control conditions in a hydroponic culture system. Data shows mean ± s.e. of at least 4 biological replicates relative to the control (24 h, 0 μM CdSO_4_). The letters a-d represent groups with a significantly different gene expression (Tukey’s test: p < 0.05). Statistics was performed separately within each exposure time.

In roots, Cd exerted the greatest effect on the expression of all three genes after 24 h of exposure. The expression of *ACO2* was significantly higher in wild-type plants as compared to the mutants after 24 h exposure to both concentrations. However, after 72 h of Cd exposure there were no significant differences between wild-type and mutant plants (Figure 5A). For *ETR2* expression, a similar pattern as for *ACO2* was observed, except after 24 h exposure to 10 μM Cd, no significant differences between both genotypes were observed (Figure 5C). The expression of *ERF1* in the wildtype was significantly higher compared to the mutant after exposure to 5 μM Cd, and after 72 h of exposure to 10 μM Cd (Figure 5E).

In leaves, the expression of these three genes was always significantly higher in wild-type plants after 24 h exposure to both Cd concentrations compared to the mutants (Figure 5B,D,F). After 72 h of exposure there were less significant differences, only *ACO2* showed significantly higher transcript levels in wild-type plants compared to the mutants exposed to 5 μM Cd (Figure 5B,D,F).

## Discussion

### Cadmium stress increases ethylene production in *Arabidopsis thaliana*

Ethylene is a well-known regulator of miscellaneous plant responses, and is affected by many biotic and abiotic stresses [17],[21],[43]. Also after exposure to excess metals, increased ethylene levels have been observed [15],[34],[36],[38],[39]. Ethylene is enzymatically synthesised from SAM in two steps, with ACS, encoded by a multigene family, as the rate-limiting enzyme [21],[22]. Still, it remains unclear how an increase in ethylene release after toxic metal exposure is achieved at the molecular level. Therefore, in the present study, a kinetic approach was adopted to investigate the effects of Cd exposure on ACC and ethylene production in *Arabidopsis thaliana* as well as the influence of Cd on the expression of the *ACS* and *ACO* multigene families involved in ethylene biosynthesis.

The immediate precursor of ethylene, ACC, exists in a free (active) as well as conjugated (inactive) form. Although being reversible to a certain extent, the conjugation of ACC makes it, at least temporarily, unavailable for the ethylene biosynthesis pathway [41]. The accumulation of conjugated ACC could serve to optimise free ACC levels as a substrate for ACO, converting it to ethylene. Deconjugation can subsequently restore free ACC levels to avoid depletion. In contrast with previous studies, we quantified both forms of ACC separately, not only focussing on free ACC. Exposure to 5 or 10 μM Cd induced the accumulation of free as well as conjugated ACC in roots and leaves of wild-type *Arabidopsis thaliana* plants grown in hydroponics (Figure 1). This can explain the observed increase in ethylene release under Cd stress (Figure 3). In roots, the overall ACC content is lower compared to leaves. This could be due to a lower production rate or transportation of ACC from the roots to the leaves [44]. The fact that exposure to 5 μM Cd did not significantly increase the ACC content in roots could be explained by the rate-limiting character of this step. Most of the ACC could immediately be converted into ethylene, as observed from the ethylene biosynthesis data (Figure 3). This hypothesis can also be confirmed by the increase in expression of ethylene responsive genes in roots after exposure to 5 μM Cd (Figure 5A, C,E). Previous studies also reported increasing ACC contents in roots and leaves of tomato plants after three weeks of growth on salinised medium [45]-[47]. Likewise, Siddikee et al. [48] observed higher ACC levels in roots of two weeks old red pepper plants exposed to salt stress for one week. On the contrary, Ben Salah et al. [49] reported a decrease in ACC content after 3 weeks of salt stress in roots and leaves of the salt-tolerant *Medicago ciliaris*. In contrast with our findings, Han et al. [50] did not find a clear correlation between Cd exposure and ACC content in leaves of the halophyte *Kosteletzkya virginica*. Three weeks of exposure to 5 μM Cd did not increase the ACC concentration, addition of 50 mM NaCl together with Cd even decreased the ACC content. This points to different responses in salt tolerant and sensitive species.

Genes known to be responsive to elevated ethylene levels showed an increase in expression in hydroponically cultivated plants exposed to 5 or 10 μM Cd (Figure 5), also indicating an augmentation of ethylene biosynthesis. The latter was verified in our study in wild-type plants grown on rockwool, displaying a dose-dependent increase in ethylene production after 24 and 72 h of exposure to 10, 25 and 100 μM Cd (Figure 3). Consequently both the hydroponic and rockwool growth system clearly support a Cd-induced ethylene biosynthesis.

### The stress related ACS2 and ACS6 are the main isoforms involved in Cd-induced ethylene production

To further unravel these findings, the expression of genes encoding the enzymes involved in ethylene biosynthesis, ACS and ACO were analysed. Hitherto, few studies investigated the effect of toxic metals on the differential expression of the *ACO* multigene family members. Srivastava et al. [51] reported a lead-induced upregulation of a putative *ACO* gene in *Sesbania drummondii*. Kim et al. [52] observed increased *ACO1* and *ACO3* transcript levels in *Nicotiana glutinosa* after 48 h of exposure to Cu. Dorling et al. [53] on the other hand did not detect differences in *ACO* transcript levels of *Trifolium repens* after 9 days of excess manganese (Mn). To the best of our knowledge, this is the first time the effect of toxic sublethal Cd exposure on *ACO* gene expression was investigated in *Arabidopsis thaliana*. The transcript levels of *ACO2* and *ACO4*, the two most abundant members of the *ACO* multigene family, coding for the enzymes responsible for the conversion of ACC to ethylene (Figure 2C and D) increased in a dose-dependent manner. These results corroborate the conclusions of Ruduś et al. [54], who observed upregulations of various *ACO* genes after exposure to abiotic (wounding, flooding) and biotic (pathological infection) stresses, serves as a good ethylene production indicator.

The rate-limiting step in ethylene biosynthesis, however, is the conversion of SAM to ACC by ACS [21]. The expression of eight different genes coding for the ACS isoforms was assessed (Figure 2A and B). The maximum increases in expression of *ACS* genes, after 72 h or 24 h of exposure to Cd for respectively roots and leaves, correlated well with the ACC content in both organs (Figure 1). Cadmium exposure particularly increased the abundance of *ACS2* and *ACS6* transcript levels. These two isoforms are the only active type 1 ACS proteins, making them phosphorylation targets of mitogen-activating protein kinase (MAPK) MPK3/MPK6. This posttranslational modification reduces the turnover by the 26S proteasome degradation machinery, prolonging the half-life of the ACS enzymes [21],[55],[56]. In addition, MPK3 and MPK6 are also capable of inducing the transcriptional activity of *ACS2* and *ACS6* via WRKY33 [57]. The involvement of MAPK signalling in plants under metal stress has been reported several times [58]. Jonak et al. [59] showed that SAMK/SIMK, the *Arabidopsis* orthologues of MPK3/MPK6 in *Medicago sativa*, were activated after exposure to excess Cd or Cu ions. In *Arabidopsis thaliana*, MPK3/MPK6 activity and mRNA levels were also induced after exposure to Cd [58],[60],[61]. Various other abiotic stresses are also known to elevate ethylene biosynthesis through induction of different *ACS* transcript levels in *Arabidopsis thaliana*[20]. Interestingly, ACS2 and ACS6 very often appear to regulate the production of stress ethylene in *Arabidopsis thaliana. ACS6* transcript levels were shown to be elevated after exposure to ozone, Li (lithium), Cu, salt stress, … [62],[63]. *ACS2* gene expression was also upregulated by high salinity [64]. Peng et al. [65] reported the induction of *ACS2* and *ACS6* up to 36 h of hypoxic treatments. In addition, the necrotrophic fungus *Botrytis cinerea* is known to induce ethylene production through an ACS2 and ACS6 dependent mechanism [57],[66].

In this study, evidence for the importance of *ACS2* and *ACS6* upregulation in Cd-induced ethylene production was found using the *Arabidopsis thaliana acs2-1acs6-1* double KO-mutant, which showed a much lower induction of ethylene production. The basal level of ethylene production measured in these mutants may be explained by the presence of other ACS isoforms, which, because of their minor abundance after Cd exposure at transcriptional (except for *ACS7*) or protein level (Additional file 2), gave rise to low ethylene levels. Many of these other isoforms have been reported to be involved in developmental regulation, rather than stress [67]-[69].

No significant differences were found in Cd content between wild-type and mutant plants, indicating that the absence of induction of ethylene production in mutants was not attributable to a decreased Cd uptake (Table 1).

With the objective to investigate the consequences for signalling and perception of the lack of ethylene biosynthesis induction, the physiological responses as well as the expression of ethylene responsive genes were measured in *acs2-1acs6-1* mutants and compared to wild-type plants.

As mentioned, no significant differences in root fresh weight were observed between both genotypes (Figure 4A). In leaves however, Cd induced a significant growth inhibition in the wild-type but not or to a lesser extent in the mutant plants, more specifically at 24 h and 72 h for 10 and 5 μM Cd respectively. This was also reflected in the fresh weight data (Figure 4B). Hence, within our experimental setup, the negatively affected leaf biomass in wild-type plants was a consequence of Cd-induced ethylene production.

The ethylene biosynthesis gene *ACO2*, the ethylene receptor gene *ETR2* and the ethylene response factor gene *ERF1* are known to have elevated transcript levels in response to ethylene exposure [70]-[73]. ERF1 is also known to be involved in different stress responses. Cheng et al. [74] reported that the induction of *ERF1* gene expression after salt and dehydration stress was enhanced by ethylene signalling. Therefore we assumed *ERF1* to be the most indicative ethylene responsive gene of our selection. After exposure of our plants to Cd, the expression of the three genes was, as mentioned before, significantly higher in roots and leaves of wild-type plants. In the mutants, however, there was evidence for a lower induction of expression of the ethylene responsive genes (Figure 5). The remaining elevated transcript levels of these genes in the roots of mutant plants, especially after exposure to 10 μM Cd, can be explained by the increase in expression of *ACS7*, possibly leading to increased ethylene release (Figure 2A, Additional file 2). After 72 h of exposure to Cd the differences in ethylene responsive gene expression between the two genotypes started to fade. Except for the expression of *ACO2* and *ETR2* in the leaves, the transcript levels of the ethylene responsive genes decreased compared to 24 h of exposure to Cd. This could be caused by a transient response of the genes to the ethylene signal, indicating the importance of ethylene in the early response to Cd stress. These results correspond to those of Montero-Palmero et al. [19], who also observed a transient induction of ethylene responses in mercury (Hg) treated *Medicago sativa* and *Arabidopsis thaliana* seedlings. The increased *ACO2* and *ETR2* expression in the leaves of both genotypes after 72 h of exposure to Cd could be the result of Cd-induced signalling pathways independent of ACS2 and ACS6.

## Conclusions

In conclusion, Cd induced the biosynthesis of ACC and ethylene in *Arabidopsis thaliana* plants mainly via the increased expression of *ACS2* and *ACS6*, which was confirmed by the low ethylene levels in *acs2-1acs6-1* double KO-mutants exposed to Cd. Whereas other isoforms still deliver a basal ethylene level, the lack of Cd-induced increase in ethylene production in the double mutants highly diminished the fast-induced expression of ethylene responsive genes, which positively affected the plant leaf biomass.

## Methods

### Plant material, culture and treatment

*Arabidopsis thaliana* (Columbia ecotype) wild-type and *acs2-1acs6-1* double KO-mutant seeds (N16581) were obtained from the European Arabidopsis Stock Centre (NASC). These mutant plants were described by Tsuchisaka et al. [75] and they were checked for homozygosity by PCR as instructed.

After surface sterilisation, seedlings were cultivated using a modified Hoagland nutrient solution either (1) on hydroponics according to Smeets et al. [76], but using purified sand or (2) on rockwool plugs. Established growth conditions for both culturing systems were 12 h photoperiod with day/night temperatures of respectively 22/18°C and 65% relative humidity. A combination of blue, red and far-red led modules (Philips Green-Power LED modules) was used to simulate the photosynthetic active radiation (PAR) spectrum of sunlight with a photosynthetic photon flux density of 170 μmol m^−1^ s^−1^ at the leaf level [77].

Three weeks old plants grown on hydroponics were exposed to 5 or 10 μM CdSO_4_ at the root level (except for control plants). These sublethal concentrations are commonly found in the pore water of moderately contaminated soils and were also applied in previous hydroponic growth experiments [78]. After 24 or 72 h of exposure, whole root and shoot systems were separated, sampled and snap frozen in liquid nitrogen prior to storage at −70°C and further analyses except for quantification of Cd contents. Biological replicates for each measured parameter (number of replicates displayed in table and figure legends) were sampled from various pots of the same conditions to avoid within pot correlation [76].

For ethylene emission analysis using the rockwool (Grodan Delta, Grodan, Roermond, The Netherlands) cultivation system, seven plants were grown per plug (5 cm diameter, 3.5 cm height), pre-moistened with the same modified Hoagland nutrient solution as in hydroponics. The plugs were positioned in modified Aratrays (Arasystem, Beta Tech, Ghent, Belgium) and placed in lightproof containers filled with 1 L modified Hoagland nutrient solution, leaving only the surface of the plugs, and later the shoots of the plants visible (Additional file 3). The nutrient solution was refreshed twice a week.

### Quantification of Cd contents

Roots and leaves of hydroponically grown plants were harvested. Roots were washed for 15 min with ice-cold 10 mM Pb(NO_3_)_2_ and rinsed in distilled water at 4°C to exchange surface-bound elements [79]. Leaves were rinsed with distilled water. Samples were oven-dried at 80°C and digested in HNO_3_ (70–71%) in a heat block. Cadmium concentrations in the extracts were determined by inductively coupled plasma-atomic emission spectrometry (ICP-AES, Perkin-Elmer, 1100B, USA). As references, blanks (HNO_3_ only) and certified standard samples (NIST Spinach (1570a)) were analysed. For rockwool-cultivated plants, leaves were processed identically. In this system, roots were not freely available and could therefore not be analysed.

### Determination of ACC content

Root and leaf samples of hydroponically grown plants were ground under frozen conditions in a Retsch Mixer Mill 2000 (Retsch, Haan, Germany) using stainless steel beads. D_4_-ACC (250 pmol, Olchemim Ltd. Olomouc, CZ. Rep.) was added as internal standard for quantification. ACC was extracted by a solid-phase extraction procedure using half the extract [80]. ACC-conjugates were purified and analysed as ACC after dry acid hydrolysis of the second half of the extract [81]. Subsequently, both fractions were derivatised with pentafluorobenzyl (PFB) bromide and analysed as PFB-bis-ACC by Negative Ion Chemical Ionisation Gas chromatography–mass spectrometry (NICI GC-MS) following Smets et al. [80] (Quattro micro MS/MS, Waters, Manchester, UK, E.E. 70 eV, Emission 200 μA, extraction 10 V, Source 206 μA, GC interface T: 120°C, CI gas flow 69 mL/min, WCOT CP-Sil 5 C8 Low bleed/MS column, 30 m, 250 μm, film thickness 0.25 μm (Varian), mobile phase helium, T gradient 50 to 250°C at 25°C/min) [82]. The diagnostic transitions used for Multiple Reaction Monitoring (MRM) were for ACC: 280 > 112 and 280 > 167 and for D4-ACC: 284 > 116 and 284 > 167 corresponding to their pentafluorobenzyl (PFB-bis-ACC) derivatives. The transitions 280 > 114 and 284 > 116 were used for calculating concentrations. Data are expressed in picomoles per milligram fresh weight (pmol mg^−1^ FW^−1^).

### Gene expression analysis

From root and leaf tissues of hydroponically grown plants, disrupted the same way as for the ACC content, RNA was extracted using the RNAqueous® Phenol-free total RNA Isolation Kit (Ambion, Life Technologies, Paisley, UK), according to the manufacturers instructions. RNA concentration and purity was evaluated spectrophotometrically on the NanoDrop ND-1000 (ThermoScientific, Wilmington, DE, USA). DNase treatment with the TURBO DNA-free™ Kit (Ambion, Life Technologies, Paisley, UK) was performed to eliminate possible genomic DNA contamination. One μg of the treated RNA per sample was converted to single stranded cDNA using the High-Capacity cDNA Reverse Transcription Kit (Ambion, Life Technologies, Paisley, UK) according to the manufacturers instructions. A 10-fold dilution of the produced cDNA was prepared in 1/10 diluted TE buffer (1 mM Tris–HCl, 0.1 mM Na2-EDTA, pH 8.0; Sigma–Aldrich, Belgium) and stored at −20°C. Quantitative real-time PCR was performed in an optical 96-well plate with the 7900HT Fast Real-Time PCR System (Life Technologies, Paisley, UK) using SYBR Green chemistry. Gene-specific forward and reverse primers were designed and optimised via the Primer Express software (v2.0, Life Technologies, Paisley, UK). Amplification occurred at universal cycling conditions (20 s at 95°C, 40 cycles of 1 s at 95°C and 20 s at 60°C) followed by the generation of a dissociation curve to verify amplification specificity. Reactions contained 2 μL diluted cDNA template (or RNase-free H_2_O for the ‘no template controls’), 5 μL 2× Fast SYBR® Green Master Mix (Life Technologies, Paisley, UK), forward and reverse primers (300 nM each, unless otherwise mentioned in Additional file 4) and 2.4 μL RNase-free H_2_O in a total volume of 10 μL. The specificity of the used primer pairs was checked *in silico* using Blast (http://www.arabidopsis.org/Blast/index.jsp) and after qPCR by verifying single peaks on the dissociation curve. In addition, primer efficiency (e) was evaluated on a standard curve generated using a twofold dilution series of a mixed sample over at least five dilution points and verified to be higher than 80% (e = 10^(−1/slope)). In Additional file 4, all gene annotations, primer sequences and primer efficiencies are shown. Gene expression levels were calculated according to the e^−ΔCq^ method relative to the sample with the highest expression (minimum Cq). The data obtained were normalised using the geometric average of the 2^−ΔCq^ values of three stable reference genes selected out of a set of 10 [83] by geNorm (v3.5) and Normfinder (v0.953) algorithms [84],[85]. According to the experimental set-up the most stable reference genes were used to determine sample-specific normalisation factors (Additional file 5).

To calculate the relative abundance of distinct gene family members, the expression level of each family member was determined for the control sample panel (0 h, 0 μM Cd) relative to the highest expressed family member. This yields a relative abundance factor for each member of the gene family, which is used in the calculation of its relative abundance in the kinetic Cd exposure experimental setup. Subsequently, the changes in expression level for each member of a gene family were determined in function of the exposure time and Cd concentration applied and set relatively to the control (0 h, 0 μM Cd).

### Determination of ethylene production

Rockwool plugs containing three weeks old plants or blank plugs as mock controls were individually transferred into closed glass cuvettes (7 cm in diameter, 7 cm high) kept at 12/12 light/dark regime and exposed at dawn to 0, 10, 25 or 100 μM CdSO_4_ by injection in the middle of the rockwool plug. The cuvettes were flushed with hydrocarbon free air (Air Liquide, Aalter, Belgium) every 24 h. The ethylene in the headspace was detected by an ETD-300 Photo-acoustic ethylene detection system (Sensor Sense, Nijmegen, The Netherlands) and analysed using microcal Origin software (Northampton, Massachusetts). Ethylene standard mixtures for calibration were supplied by AirLiquide. Ethylene production was calculated in picolitres per milligram fresh weight per hour (pL mg^−1^ FW^−1^ h^−1^).

### Statistical analysis

The datasets were analysed via the linear model procedure in R [86]. Both normality (Shapiro-Wilk test) and homoscedasticity (residue plot) were checked; transformations were applied when necessary to approximate normality. Normally distributed data were analysed using the one- or two-way ANOVA procedure. Tukey–Kramer adjustment for multiple comparisons was applied to obtain corrected p-values. The statistical analyses of non-normally distributed data were based on the non-parametric Kruskal–Wallis test followed by the post hoc pairwise Wilcoxon rank sum test.

## Competing interests

The authors declare that they have no competing interests.

## Authors’ contributions

KS, DVDS, FV, TR, JV, AC participated in the conception of the study and the design of the experiments. KS performed most of the experiments and wrote the manuscript. TR and AC assisted with writing the manuscript. DVDS and FV performed the ethylene measurements and analyses. EP assisted with the ACC measurements and analyses. All authors read and approved the final manuscript after critical revision.

## Additional files

## Supplementary Material

Additional file 1: **Relative expression of*ACC oxidase* and *ACC synthase* genes.** Relative expression of ACC oxidase and ACC synthase genes in roots (A) and leaves (B) of 3 weeks old *Arabidopsis thaliana* plants exposed for 24 or 72 h to either 5 or 10 μM CdSO_4_ or grown under control conditions in a hydroponic culture system. Data shows mean ± s.e. of at least 4 biological replicates relative to the control within each time point. The colours represent groups with a significantly different expression (green: decrease; red: increase; Tukey’s test: p < 0.05). Statistics was performed separately for each gene within each exposure time. Click here for file

Additional file 2: **Relative expression of*ACS7.*** Relative expression of *ACS7* in roots and leaves of 3 weeks old wild-type or *acs2-1acs6-1* mutant *Arabidopsis thaliana* plants exposed for 24 or 72 h to either 5 or 10 μM CdSO_4_ or grown under control conditions in a hydroponic culture system. Data shows mean ± s.e. of at least 4 biological replicates relative to the control (24 h, 0 μM CdSO_4_). The letters a-d represent groups with a significantly different gene expression (Tukey’s test: p < 0.05). Statistics was performed separately within each exposure time. Click here for file

Additional file 3: **Rockwool cultivation system.** (A) 7 *Arabidopsis thaliana* plants sown on rockwool covered with aluminium foil, positioned in modified Aratrays and placed in lightproof containers filled with 1 L modified Hoagland nutrient solution, leaving only the surface of the plugs visible. (B) Rockwool plugs containing three weeks old plants were transferred to glass cuvettes and connected to the measurement system (the aluminium foil was removed). Click here for file

Additional file 4: **Primer and amplicon information.** Primer and amplicon information (*not measurable by dilution series due to extremely low expression). Click here for file

Additional file 5: Reference gene information. Click here for file
