# Supplementary material for: Cadmium-induced ethylene production and responses in Arabidopsis thaliana rely on ACS2 and ACS6 gene expression
Source: BMC Plant Biol. 2014 Aug 1;14:214. doi: 10.1186/s12870-014-0214-6 (PMC4236733; doi:10.1186/s12870-014-0214-6)
Supplement: Additional file 1: — Relative expression ofACC oxidase and ACC synthase genes. Relative expression of ACC oxidase and ACC synthase genes in roots (A) and leaves (B) of 3 weeks old Arabidopsis thaliana plants exposed for 24 or 72 h to either 5 or 10 μM CdSO4 or grown under control conditions in a hydroponic culture system. Data shows mean ± s.e. of at least 4 biological replicates relative to the control within each time point. The colours represent groups with a significantly different expression (green: decrease; red: increase; Tukey’s test: p < 0.05). Statistics was performed separately for each gene within each exposure time. [file s12870-014-0214-6-S1.zip › 1859074107128928_MOESM5_ESM.pdf]

A

Roots — ***ACC Oxidase***

Gene	$CdSO_4$ (µM)	0 h			24 h			72 h		
ACO1	0	1.00	±	0.06	1.00	±	0.11	1.00	±	0.07
	5				0.90	±	0.10	0.79	±	0.10
	10				0.55	±	0.14	0.38	±	0.05
ACO2	0	1.00	±	0.10	1.00	±	0.05	1.00	±	0.11
	5				1.32	±	0.14	0.88	±	0.15
	10				3.00	±	0.34	1.65	±	0.39
ACO-like (AT1G77330)	0	1.00	±	0.04	1.00	±	0.05	1.00	±	0.07
	5				2.08	±	0.24	0.79	±	0.08
	10				2.03	±	0.30	0.48	±	0.06
ACO4	0	1.00	±	0.10	1.00	±	0.10	1.00	±	0.08
	5				2.75	±	0.22	2.83	±	0.35
	10				8.47	±	1.00	7.04	±	1.50
ACO-like (AT1G12010)	0	1.00	±	0.09	1.00	±	0.12	1.00	±	0.12
	5				1.17	±	0.23	1.51	±	0.04
	10				2.18	±	0.40	1.70	±	0.20

ACC Synthase

Gene	$CdSO_4$ (µM)	0 h			24 h			72 h		
ACS1	0	1.00	±	0.08	1.00	±	0.28	1.00	±	0.18
	5				0.99	±	0.25	0.92	±	0.10
	10				1.33	±	0.47	30.34	±	12.11
ACS2	0	1.00	±	0.09	1.00	±	0.05	1.00	±	0.04
	5				1.44	±	0.56	1.23	±	0.39
	10				7.93	±	2.93	35.13	±	10.73
ACS4	0	1.00	±	0.15	1.00	±	0.15	1.00	±	0.34
	5				1.28	±	0.39	1.44	±	0.58
	10				2.01	±	0.14	1.36	±	0.33
ACS5	0	1.00	±	0.08	1.00	±	0.13	1.00	±	0.08
	5				0.10	±	0.05	0.10	±	0.05
	10				0.02	±	0.01	0.01	±	0.00
ACS6	0	1.00	±	0.13	1.00	±	0.08	1.00	±	0.07
	5				2.35	±	0.37	1.73	±	0.08
	10				8.17	±	1.69	9.74	±	2.00
ACS7	0	1.00	±	0.11	1.00	±	0.32	1.00	±	0.17
	5				1.90	±	0.27	3.28	±	0.93
	10				10.07	±	1.11	35.63	±	4.28
ACS8	0	1.00	±	0.14	1.00	±	0.23	1.00	±	0.22
	5				2.67	±	1.28	1.69	±	0.29
	10				22.83	±	7.71	35.40	±	10.74
ACS11	0	1.00	±	0.12	1.00	±	0.22	1.00	±	0.13
	5				3.76	±	0.64	1.67	±	0.17
	10				5.27	±	1.31	1.65	±	0.69
