# Supplementary material for: Cadmium-induced ethylene production and responses in Arabidopsis thaliana rely on ACS2 and ACS6 gene expression
Source: BMC Plant Biol. 2014 Aug 1;14:214. doi: 10.1186/s12870-014-0214-6 (PMC4236733; doi:10.1186/s12870-014-0214-6)
Supplement: Additional file 1: — Relative expression ofACC oxidase and ACC synthase genes. Relative expression of ACC oxidase and ACC synthase genes in roots (A) and leaves (B) of 3 weeks old Arabidopsis thaliana plants exposed for 24 or 72 h to either 5 or 10 μM CdSO4 or grown under control conditions in a hydroponic culture system. Data shows mean ± s.e. of at least 4 biological replicates relative to the control within each time point. The colours represent groups with a significantly different expression (green: decrease; red: increase; Tukey’s test: p < 0.05). Statistics was performed separately for each gene within each exposure time. [file s12870-014-0214-6-S1.zip › 1859074107128928_MOESM6_ESM.pdf]

**B**

*Leaves* — *ACC Oxidase*

| Gene | CdSO$_4$ (µM) | 0 h | | | 24 h | | | 72 h | | |
|---|---|---|---|---|---|---|---|---|---|---|
| ***ACO1*** | 0 | 1.00 | ± | 0.19 | 1.00 | ± | 0.22 | 1.00 | ± | 0.24 |
| | 5 | | | | 0.42 | ± | 0.09 | 0.54 | ± | 0.15 |
| | 10 | | | | 0.34 | ± | 0.09 | 0.46 | ± | 0.08 |
| ***ACO2*** | 0 | 1.00 | ± | 0.06 | 1.00 | ± | 0.06 | 1.00 | ± | 0.08 |
| | 5 | | | | 1.88 | ± | 0.48 | 1.53 | ± | 0.10 |
| | 10 | | | | 3.98 | ± | 0.71 | 5.67 | ± | 0.84 |
| ***ACO-like (AT1G77330)*** | 0 | 1.00 | ± | 0.06 | 1.00 | ± | 0.08 | 1.00 | ± | 0.06 |
| | 5 | | | | 0.52 | ± | 0.12 | 0.68 | ± | 0.25 |
| | 10 | | | | 0.51 | ± | 0.16 | 0.42 | ± | 0.13 |
| ***ACO4*** | 0 | 1.00 | ± | 0.07 | 1.00 | ± | 0.02 | 1.00 | ± | 0.12 |
| | 5 | | | | 7.17 | ± | 1.41 | 2.57 | ± | 0.33 |
| | 10 | | | | 10.07 | ± | 1.30 | 3.56 | ± | 0.27 |
| ***ACO-like (AT1G12010)*** | 0 | 1.00 | ± | 0.05 | 1.00 | ± | 0.02 | 1.00 | ± | 0.01 |
| | 5 | | | | 0.43 | ± | 0.12 | 0.60 | ± | 0.20 |
| | 10 | | | | 0.29 | ± | 0.05 | 0.26 | ± | 0.04 |

*ACC Synthase*

| Gene | CdSO$_4$ (µM) | 0 h | | | 24 h | | | 72 h | | |
|---|---|---|---|---|---|---|---|---|---|---|
| ***ACS1*** | 0 | 1.00 | ± | 0.09 | 1.00 | ± | 0.14 | 1.00 | ± | 0.21 |
| | 5 | | | | 1.38 | ± | 0.40 | 1.02 | ± | 0.26 |
| | 10 | | | | 2.50 | ± | 0.57 | 3.75 | ± | 0.86 |
| ***ACS2*** | 0 | 1.00 | ± | 0.13 | 1.00 | ± | 0.04 | 1.00 | ± | 0.39 |
| | 5 | | | | 538.47 | ± | 156.38 | 27.26 | ± | 12.67 |
| | 10 | | | | 2043.65 | ± | 655.14 | 254.89 | ± | 79.48 |
| ***ACS4*** | 0 | 1.00 | ± | 0.19 | 1.00 | ± | 0.20 | 1.00 | ± | 0.24 |
| | 5 | | | | 0.09 | ± | 0.00 | 0.30 | ± | 0.08 |
| | 10 | | | | 0.04 | ± | 0.03 | 0.03 | ± | 0.01 |
| ***ACS5*** | 0 | 1.00 | ± | 0.16 | 1.00 | ± | 0.20 | 1.00 | ± | 0.30 |
| | 5 | | | | 0.03 | ± | 0.01 | 0.11 | ± | 0.03 |
| | 10 | | | | 0.07 | ± | 0.04 | 0.10 | ± | - |
| ***ACS6*** | 0 | 1.00 | ± | 0.07 | 1.00 | ± | 0.11 | 1.00 | ± | 0.17 |
| | 5 | | | | 11.76 | ± | 2.82 | 2.24 | ± | 0.63 |
| | 10 | | | | 20.70 | ± | 2.92 | 2.60 | ± | 0.32 |
| ***ACS7*** | 0 | 1.00 | ± | 0.05 | 1.00 | ± | 0.11 | 1.00 | ± | 0.15 |
| | 5 | | | | 2.26 | ± | 0.29 | 1.73 | ± | 0.65 |
| | 10 | | | | 5.43 | ± | 1.34 | 6.81 | ± | 1.15 |
| ***ACS8*** | 0 | 1.00 | ± | 0.14 | 1.00 | ± | 0.26 | 1.00 | ± | 0.19 |
| | 5 | | | | 6.93 | ± | 1.93 | 2.16 | ± | 0.64 |
| | 10 | | | | 8.04 | ± | 3.08 | 2.50 | ± | 0.44 |
| ***ACS11*** | 0 | 1.00 | ± | 0.16 | 1.00 | ± | 0.12 | 1.00 | ± | 0.04 |
| | 5 | | | | 1.50 | ± | 0.33 | 1.49 | ± | 0.57 |
| | 10 | | | | 1.76 | ± | 0.47 | 1.09 | ± | 0.66 |
