# Supplementary figures and images for: Cadmium-induced ethylene production and responses in Arabidopsis thaliana rely on ACS2 and ACS6 gene expression
Source: BMC Plant Biol. 2014 Aug 1;14:214. doi: 10.1186/s12870-014-0214-6 (PMC4236733; doi:10.1186/s12870-014-0214-6)

**ACS7: Roots**

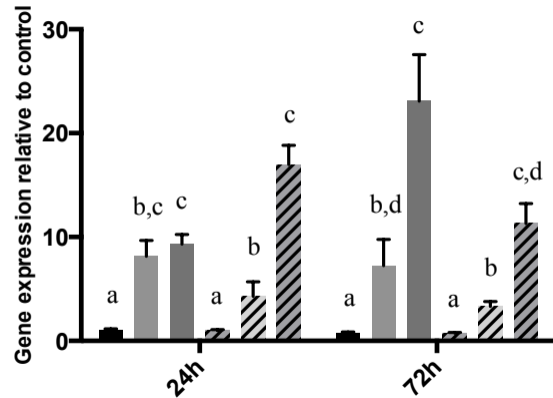

**ACS7: Leaves**

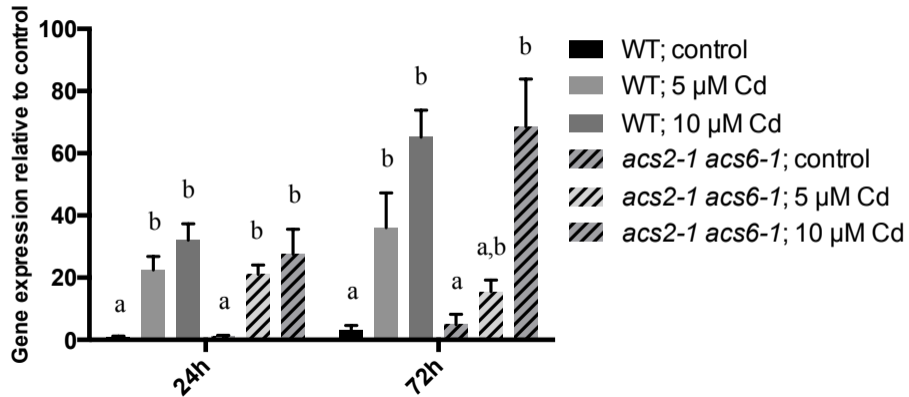

Supplement: Additional file 2: — Relative expression ofACS7. Relative expression of ACS7 in roots and leaves of 3 weeks old wild-type or acs2-1acs6-1 mutant Arabidopsis thaliana plants exposed for 24 or 72 h to either 5 or 10 μM CdSO4 or grown under control conditions in a hydroponic culture system. Data shows mean ± s.e. of at least 4 biological replicates relative to the control (24 h, 0 μM CdSO4). The letters a-d represent groups with a significantly different gene expression (Tukey’s test: p < 0.05). Statistics was performed separately within each exposure time. [file s12870-014-0214-6-S2.pdf]

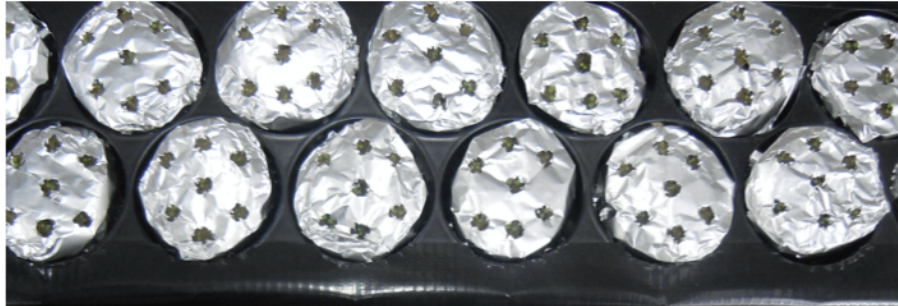

A

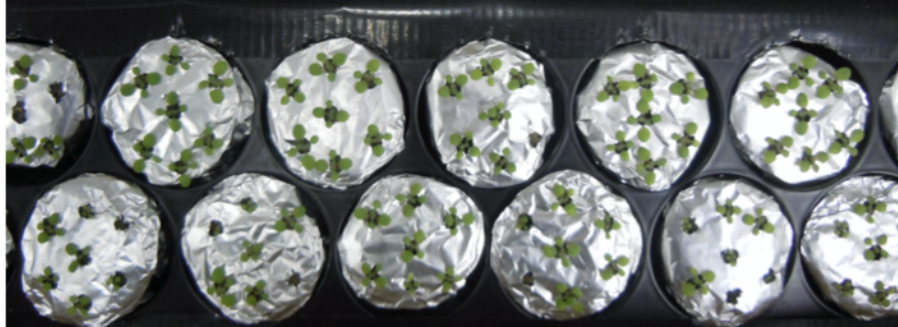

B

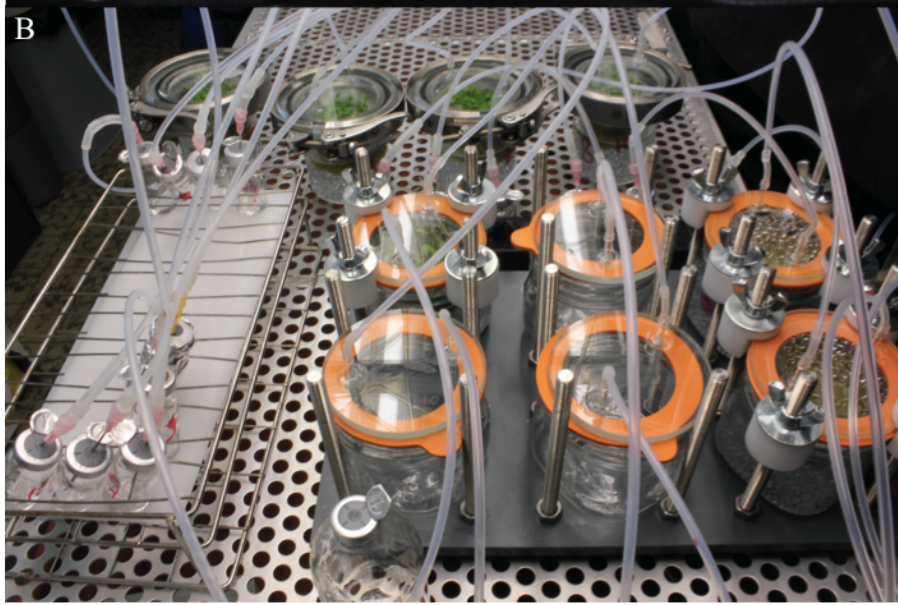

Supplement: Additional file 3: — Rockwool cultivation system. (A) 7 Arabidopsis thaliana plants sown on rockwool covered with aluminium foil, positioned in modified Aratrays and placed in lightproof containers filled with 1 L modified Hoagland nutrient solution, leaving only the surface of the plugs visible. (B) Rockwool plugs containing three weeks old plants were transferred to glass cuvettes and connected to the measurement system (the aluminium foil was removed). [file s12870-014-0214-6-S3.pdf]
